# Tests to uncover and assess breathlessness: a proposed framework

**DOI:** 10.1097/SPC.0000000000000617

**Published:** 2022-09-21

**Authors:** Magnus Ekström

**Affiliations:** Lund University, Faculty of Medicine, Department of Clinical Sciences Lund, Respiratory Medicine, Allergology and Palliative Medicine, Lund, Sweden

**Keywords:** dyspnea, exercise test, measurement, prevalence

## Abstract

Breathlessness is a common, distressing, and limiting symptom that many people avoid by reducing their activity. This review discusses exertional tests that can be used for uncovering and assessing breathlessness depending on the person's severity of illness, function, the setting, and aim of the assessment.

**Recent Findings:**

Standardized exertional tests are useful to uncover ‘hidden’ breathlessness earlier in people who may have adapted their physical activity to limit their breathing discomfort. In ‘more fit’ ambulatory people and outpatients, cardiopulmonary exercise testing is the gold standard for assessing symptom severity, underlying conditions, and mechanisms and treatment effects. Among field tests, the 6-min walk test is not useful for assessing breathlessness. Instead, the 3-min step test and walk test are validated for measuring breathlessness change in chronic obstructive pulmonary disease. In people with more severe illness (who are most often not breathless at rest), reported tests include upper limb exercise or counting numbers aloud, but a valid and useful test for this population is lacking.

**Summary:**

A framework for selecting the most appropriate test to assess breathlessness validly is proposed, and research needs are identified.

## INTRODUCTION

Breathlessness (breathing discomfort, dyspnea) [1] affects and limits the daily lives of millions of people worldwide, often for many years. Disabling breathlessness that persists despite best treatment – the *chronic breathlessness syndrome*[2] – is highly prevalent in people with serious life-limiting illness [3], especially in cardiopulmonary diseases. In those affected, the symptom often pervades most aspects of life [4] and becomes more distressing as death approaches [5].

Breathlessness often remains ‘hidden’ [6], and is challenging to quantify accurately. *‘How is your breathing?’* and similar questions that are commonly used in clinical care (including by the author) are insufficient to reliably uncover the presence of the symptom or capture its severity. Asking people to grade the severity of their symptoms on a scale [such as a visual analog scale (VAS) or numerical rating scale (NRS)] is important as patients frequently do not report symptoms spontaneously. However, in many settings using a validated rating scale is not enough, for several important reasons. First, such ratings most often ask the person to recall symptoms during a time period (such as the last 24 h). Recalled symptoms are influence by multiple factors, such as cognition, memory, peak, and recent symptom levels (’peak-end-rule’) [7], setting, and circumstances and can differ substantially from actual experienced ratings during the same period [8^▪▪^]. Second, the symptom report is affected (confounded) by the level of physical activity. Even people with severe illness can reduce their breathing distress by becoming more inactive, creating a vicious breathlessness-deconditioning-cycle [9]. Few people, even with severe illness, are breathless at rest. Thus, ratings of breathlessness at rest or using a questionnaire of breathlessness ‘in daily life’ are likely to markedly under-estimate the presence of the symptom (‘hidden breathlessness’) [10] and its severity (due to adapted and reduced physical activity) [11,12]. In addition, symptom recall (using a question or questionnaire) is affected by multiple factors and may differ substantially from the actually experienced breathlessness [8^▪▪^,^13^]. For valid measurement, activity-related breathlessness should be quantified at a standardized level of exercise using a test [12,14].

Several tests for evaluating breathlessness are available and differ in their requirements, ease of use, target populations and settings, validity, and type of data generated. Although routine assessment of breathlessness is recommended [15], it is rarely done in clinical practice. Arguably, barriers to routine symptom assessment include the lack of a standard – or ‘toolbox’ for to assess breathlessness. Also, a suitable test to assess breathlessness in certain populations and settings may be lacking.

The aim this narrative review is to give an overview of the importance of using standardized tests for assessing breathlessness in adults, properties of available tests, and suggest a framework for deciding which test to use depending on population and setting. In addition, areas where tests are lacking and should be developed are proposed.

Relevant studies reporting on tests to evaluate exertional breathlessness were identified by searches in MEDLINE using relevant terms such as ‘test’/’evaluation’ and ‘breathlessness’/‘dyspnea’/’dyspnoea’, from database inception up to 22 June, 2022. Papers were also identified from the author's personal reference library and from the reference lists of relevant consensus, statement and review papers in this field. 

**Box 1:**
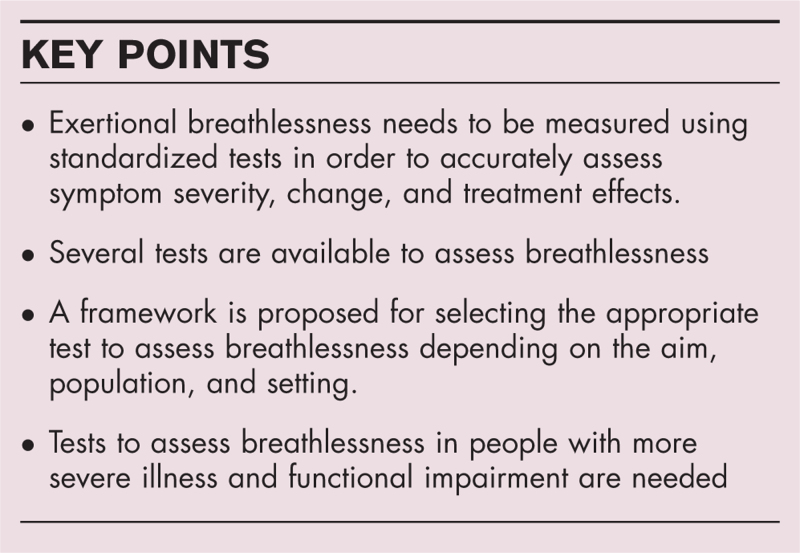
no caption available

## WHY IS STANDARDIZED TESTING NEEDED?

A central feature of breathlessness is that it can be to a large extend reduced or avoided by the person by adapting and limiting the factors triggering the symptom, mainly physical activity [16]. Activity and function is often limited by the level of breathlessness (‘symptom threshold’). An apt illustration is during symptom-limited exercise testing, such as cardiopulmonary exercise testing (CPET) or 6-min walk test, where the level of breathlessness at the end of the test (the tolerated level) is quite similar between people – and cannot even readily distinguish healthy people from those with severe disease [12,17,18]. However, the level of exertion (workload or power output) needed to provoke that symptom level differ markedly from health to people with increasing disease severity and deconditioning [19].

Advantages of assessing exertional breathlessness using a standardized test, as compared to symptom recollections at rest or during ‘daily life’, include:

(1) Uncovering under-reported or ‘hidden’ breathlessness in people with reduced activity level or function;
(2) Valid assessment of the symptom's severity – for characterization, comparing symptom levels between people, and selecting people for trials or interventions. Also, the presence and estimated severity of elevated (abnormal) breathlessness can be assessed, as well as the presence of symptom levels that are likely to be normal, by comparing the levels to those of a suitable reference material;
(3) Valid assessment of change over time or effect of treatments.

Uncovering ‘hidden’ breathlessness using exercise testing was reported by Soumagne *et al.*[11]. Among people who self-reported that they had no exertional breathlessness [defined as a modified Medical Research Council (mMRC) score of 0], those with airflow limitation (compared to healthy controls) had reduced exercise performance as well as reported markedly higher breathlessness at a given work rate or ventilation during CPET [11]. Of note, this difference was captured neither by the mMRC nor by another validated breathlessness questionnaire – the Baseline Dyspnoea Index (BDI) [11]. Thus, despite reporting no exertional breathlessness using the widely used questionnaires, the significantly worse breathlessness in people with mild chronic obsessive pulmonary disease (COPD) was uncovered when assessed using standardized exercise testing.

This is important in several ways. The global prevalence estimates that breathlessness affects around 20–25% of middle-aged and older people are based on self-ratings using mMRC. The true prevalence and severity of breathlessness across populations are therefore likely to be substantially higher and remain unexplored. This also goes for different diagnosis groups. For example, a recent large study assessed breathlessness routinely for all admissions to a tertiary care hospital during a 2-year period [20]. Of the 67 362 admissions, 23% of those admitted through the emergency department reported any breathlessness in the past 24 h, 11% percentage reported having any current breathlessness when interviewed within 12 h of admission with 4% of patients experiencing breathlessness that was rated as 4/10 or higher (which could be considered as moderate to severe). Breathlessness ≥ 4/10 was present in 43% of patients admitted with respiratory diagnoses and 25% of patients with cardiovascular diagnoses [20]. While, at first look, these findings confirm a high symptom burden among admitted patients – is it really likely that about 60% of patients admitted for respiratory disease would have only mild or even no breathlessness? If the symptom was assessed (when appropriate) in response to a standardized physical test, the true prevalence and severity of breathlessness is likely to be markedly higher.

This underreporting of breathlessness is likely to be higher in people with impaired physical activity and function, such as in advanced disease and in palliative care. More valid estimates of prevalence and severity using suitable, tailored tests are important to uncover the true burden in different populations, and to understand the full impact of breathlessness on important outcomes for the individual and for society at large.

Tests for assessing breathlessness at a standardized level of exertion can also help to correctly identify change in the symptom over time (improvement or deterioration), especially as there may be concurrent changes in physical activity and capacity, and the effect of treatments. As reviewed in more detail elsewhere [14], measurement using valid tests could potentially disentangle the conflicting findings of previous symptom studies between controlled laboratory trials and trials using ratings in daily life and establish new effective treatment to relieve this often highly distressing and limiting symptom.

## WHAT CHARACTERISES A GOOD TEST?

An optimal test to assess the severity and/or change of exertional breathlessness would meet the following proposed criteria:

(1) Standardized physical task or level of exertion (to yield a similar or predicable physiologic stimulus for the sensation)
(2) Tailored task the participant and setting (for feasibility and safety)
(3) Standardized instructions and procedures (to optimize repeatability and validity of the performance and/or ratings)
(4) Assessment of breathlessness by self-report using a validated scale (as the sensation cannot be accurately inferred from proxies or biomarkers as of yet). Suitable scales may be a Borg 0–10 (CR10) scale, 0–10 NRS, or 0–100 VAS [18].
(5) Reliability and validity for the population and setting of use
(6) Acceptable burden, requirements and costs for implementation

## WHICH TESTS ARE AVAILABLE?

Several tests are available and used to measure exertional breathlessness in different populations and settings. An overview of available tests is given in Table 1.

**Table 1:** Tests to assess exertional breathlessness

Test	Assess severity	Assess change	Advantages	Disadvantages	Comment
Laboratory					
CPET [22]	++	++	‘Golden standard’, mechanistic data	Ability to cycle/walk, costs, limited availability	Constant rate test most sensitive to evaluate change and treatment effects
Field tests					
Incremental shuttle walk test (ISWT) [24]	+	+	Require limited resources	Ability to walk	
Endurance shuttle walk test (ESWT) [24]	No	+	Require limited resources	Ability to walk. Usually need ISWT first to determine the constant speed. Duration of the test may vary considerably.	
3-min step test (3MST) [26]	(+)	++	Require limited resources	May require multiple tests for finding an appropriate speed	Need validation in other conditions than COPD
3-min walk test (3MWT) [26]	(+)	++	Require limited resources	May require multiple tests for finding an appropriate speed	Need validation in other conditions than COPD
6-min walk test (6MWT) [30]	No	No	Widely used to measure exercise capacity	Self-paced The breathlessness scores have limited discrimination and responsiveness to change	Should not be used to assess breathlessness
Arm exercise test	No	No	Feasible in more severe illness	Limited data	Not standardized or validated for measuring breathlessness
Counting–aloud tests [35]	No	No	Feasible in more severe illness	Limited ability to discriminate between severities of breathlessness	Not standardized or validated for measuring breathlessness

Usefulness rated as no, (+), + or ++ for assessing severity and change of breathlessness.

### Laboratory tests

CPET is the golden standard test to assess exertional breathlessness and can be performed using a cycle or treadmill [21,22]. Even if these exercise modalities can yield different physiological responses – with treadmill resulting in higher peak oxygen consumption (V’O_2_), more hypoxemia, more breathlessness and less leg fatigue as a cause of stopping exercise, as compared with cycle tests) – the methods result in similar breathlessness responses [23]. An incremental test is useful for stratifying symptom severity at a level of exertion as well as underlying mechanisms, while a constant work rate test is more sensitive and useful for detecting change in breathlessness and exercise endurance, such as effect of treatment [21,22]. Importantly, as for all symptom-limited tests (where peak scores are not so informative), comparing breathlessness at a similar stimulus (level of exertion or ventilation) is key.

### Field tests

Field tests to assess breathlessness include shuttle walk tests, where the person walks back and forth between markings on the level. The time (and thus, the speed) of walking between the markings is externally paced by auditory signals (‘beep tests’). First, an incremental shuttle walk tests (ISWT) is usually performed to determine the person's maximal walk speed. Then, breathlessness can be assesses using endurance tests (ESWT) performed at a constant speed (such as 75% of the maximal speed of the ISWT). The ESWT is responsive to changes in exercise endurance and breathlessness including the effect of treatment [17,24]. Breathlessness needs to be assessed at a similar time point between tests (iso-time). A potential problem is that the duration of the ESWT (and therefore, the work performed) can differ markedly between people, which makes comparisons of breathlessness severity more difficult between people, and sometimes also between time points for the same person (such as before or after an intervention).

Two breathlessness-specific field tests were recently developed – the 3-min walk test (3MWT) and 3-min step test (3MST) [25–27]. The 3MWT is a constant rate shuttle walk test between cones 9,5 m apart on the level, with a fixed duration of 3 min. In the step test, the participant steps up and down on a stair or stepping board. The speed of walking or stepping is externally paced by auditory signals. Breathlessness is measured before the test and after 1, 2 and 3 min (end). The speed of walking or stepping is determined to yield a breathlessness score of 4 or higher on a Borg CR10 scale at the end of the test (3 min), to allow either improvement or deterioration of the symptom scores. The tests have been validated in people with COPD, where both tests were responsive to change in breathlessness from bronchodilation [25,27,28]. In a recent study of 53 stable outpatients with COPD who performed a 3MST of 16 steps/min, higher breathlessness scores at 3 min predicted higher risk of a COPD exacerbation [29]. Although developed for assessing symptom change and treatment effects, this study suggests that the 3-min breathlessness tests may also be useful for stratifying breathlessness severity and risk of adverse outcomes.

Last, the 6-min walk test (6MWT) is widely used field test of exercise performance that is sometimes (incorrectly) used to assess breathlessness. It is self-paced test of walking capacity where the subject is asked to ‘walk as far as possible for six minutes’ [30]. The 6MWT often results in work that is quite intense and near the person's maximal capacity. However, as the test is self-paced, the distance walked (and work performed) differ markedly between people whereas the breathlessness scores at the end of the test are similar (commonly around 4–6 on the Borg CR10 scale), irrespective of the person's health status. The 6MWT has been found to be less responsive to change than ESWT, both for walk distance and breathlessness [17]. Therefore, the 6MWT breathlessness raw scores are not useful to evaluate breathlessness severity, change, or effect of treatment.

### More unwell patients

For people with more severe illness and disability who are not able to perform more demanding tests such as cycling, walking, or stepping, a limited number of tests have been evaluated.

Upper limb exercise tests, raising one or two arms over 40 cm, were evaluated in people with cancer and breathlessness at low levels of exertion [31]. Repeatability for breathlessness scores was best for the 2-arm test. However, data remain limited and upper limb tests are yet to be standardized or validated for measuring breathlessness.

Tests of reading numbers aloud have been evaluated, including the fifteen-count breathlessness score in 30 people with COPD [32]. The test involved taking a maximal breath in and then counting out loud to 15. While the test showed repeatability, the validation suggested insufficient ability to discriminate between people with differing severities of breathlessness [32]. Extending the test in 38 COPD patients, counting to 30, still showed insufficient discriminative ability and reading numbers aloud tests are yet to be shown useful for measuring breathlessness severity or change.

A breathlessness to maximal breathing capacity ratio during a 3-min test was evaluated in people with asthma [33]. Breathlessness was assessed using a VAS and the maximal breathing capacity by repetitive inspiratory efforts during the 3-min period. The ratio differed by the underlying asthma severity, as a marker of reduced ventilatory capacity, but the method has not been validated for measuring breathlessness *per se*.

No reports on other tests used to evaluate exercise performance, such as sit-to-stand tests, were found in terms of their utility and validity for measuring breathlessness.

The level and type of exertion needed to provoke breathlessness was evaluated in 68 people with advanced cancer [19]. With decreasing functional status, the level of exercise required as well as the proportion of people able to complete the tests decreased. The most demanding tests were in order (from hardest to easiest): the 6MWT, walking during 2 min, arm exercises, and reading numbers aloud [19]. The conclusion was that walking tests have poor utility in people with severe illness and that additional tests for measuring breathlessness in this setting are needed.

## WHICH TEST TO USE?

**FIGURE 1:**
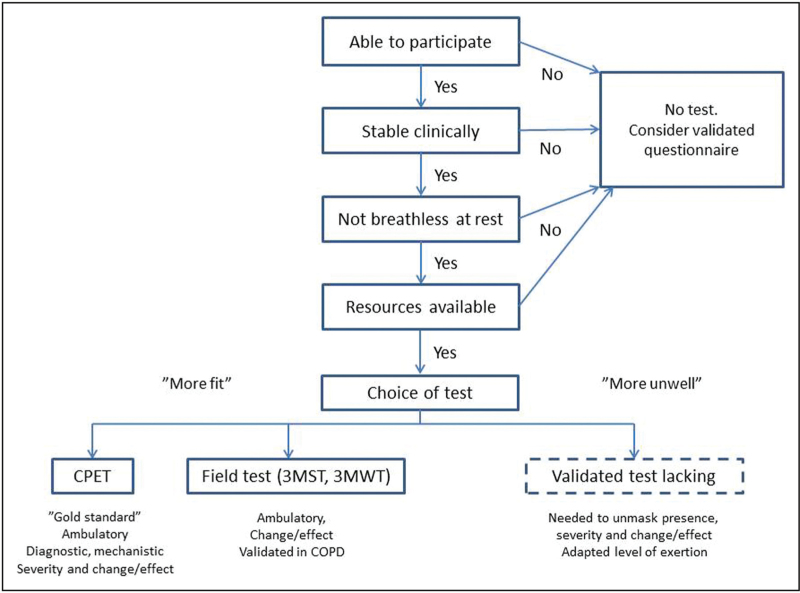
Proposed framework of available tests for assessing exertional breathlessness.

If possible, exertional breathlessness should be assessed in response to a standardized test, to account for the underlying stimulus of the symptom, such as level of exertion. The choice of which test to use will depend on several factors. A framework for selection of the most suitable test to use is proposed in Fig. 1.

The first considerations are whether the person is able and stable enough to perform a test. This clinical evaluation will both involve the current clinical state as well as recent trajectory. For example, testing in emergency department and critical care settings needs to ensure that the patient is stable and that the testing does not increase the risk of deterioration or divert attention and resources from the key management needs of the patient. The proposed next step in the framework (Fig. 1) is to identify the presence of breathlessness at rest. In people with resting breathlessness, the use of an exertion-related test is less likely to be feasible or to yield additional value above and beyond letting the patient (or proxy, when needed) rate their breathlessness at rest on a validated scale such as a 0–10 NRS [18].

In people who are able, sufficiently stable and without breathlessness at rest, the choice of test will depend on the setting, available resources and aim(s) of the assessment. As illustrated in Fig. 1, in more fit patients and especially when seeking data on underlying causes, symptom mechanisms and the effect of interventions, CPET is the gold standard for evaluating exertional breathlessness. The downside of CPET is the relatively high cost and limited availability in many settings. Alternatives for ambulatory people are field tests such as the 3MST and 3MWT, which have been validated in COPD [25–27]. The 6MWT, being a self-paced test, is not useful to assess the severity or change of breathlessness.

However, if the person is unable to walk or use a stepping board, there are currently no validated tests to uncover the presence of breathlessness and to validly measure its severity (Fig. 1).

## WHAT IS NEEDED?

As identified in this review, several developments to take forward improved measurement in relation to standardized exertional testing are needed:

(1) Reference equations for normal breathlessness responses during exercise testing, such as CPET, in healthy people, for a given level of exertion and relevant covariates such as age, sex, height and weight. Despite ranges of responses in healthy have been published [34], reference equations are lacking. Those are needed to categorize symptom severity and upper limit of normal breathlessness, for comparisons between patients and populations; ranges have been described [34], and reference equations are being developed;
(2) The breathlessness tests 3MST and 3MWT should be validated for people with conditions other than COPD, and equations to predict the right speed to obtain an appropriate breathlessness response (Borg score ≥ 4) would be helpful to reduce the number of tests needed;
(3) Methods to calculate a measure of breathlessness severity from ratings during self-paced tests such as 6MWT should be evaluated, accounting for the estimated work performed and relevant patient characteristics;
(4) Tests to evaluate severity and change in breathlessness in people with more severe illness should be developed for use at point of care, such as in the ED, at primary care visits and during hospitalizations.

## CONCLUSION

Tests are important for accurate assessment of breathlessness at a standardized level of exertion – to uncover ‘hidden’ breathlessness due to reduced physical activity, and for valid categorization of symptom severity, change over time and effect of treatment. A framework has been proposed to guide the choice of test based on factors such as the aim, population, and setting of the assessment. Tests for use in people with severe illness and reduced function are lacking, and need to be developed and validated.

## Acknowledgements

*None.*

### Financial support and sponsorship

*M.E. was supported by unrestricted grants from the Swedish Society for Medical Research and the Swedish Research Council (Dnr 2019-02081).*

### Conflicts of interest

*There are no conflicts of interest.*

## REFERENCES AND RECOMMENDED READING

Papers of particular interest, published within the annual period of review, have been highlighted as:

▪ of special interest
▪▪ of outstanding interest
